# Walking on a Balance Beam as a New Measure of Dynamic Balance to Predict Falls in Older Adults and Patients with Neurological Conditions

**DOI:** 10.1186/s40798-024-00723-7

**Published:** 2024-05-22

**Authors:** Tibor Hortobágyi, Tomas Vetrovsky, Azusa Uematsu, Lianne Sanders, Andréia Abud da Silva Costa, Rosangela Alice Batistela, Renato Moraes, Urs Granacher, Szilvia Szabó-Kóra, Bence Csutorás, Klaudia Széphelyi, József Tollár

**Affiliations:** 1 Department of Neurology, Somogy County Kaposi Mór Teaching Hospital, 7400 Kaposvár, Hungary; 2 Department of Sport Biology, Institute of Sport Sciences and Physical Education, University of Pécs, 7622 Pécs, Hungary; 3 Department of Kinesiology, Hungarian University of Sports Science, 1123 Budapest, Hungary; 4 Center for Human Movement Sciences, Medical Center, University of Groningen, University of Groningen, 9713 AV Groningen, The Netherlands; 5 Institute of Sport Research, Sports University of Tirana, Tirana, Albania; 6 Faculty of Physical Education and Sport, Charles University, Prague, Czech Republic; 7 Faculty of Sociology, Otemon Gakuin University, Ibaraki, Osaka 567-8502 Japan; 8 Lentis Center for Rehabilitation, Groningen, The Netherlands; 9 Biomechanics and Motor Control Lab, School of Physical Education and Sport of Ribeirao Preto, University of Sao Paulo, Ribeirão Preto, Brazil; 10 Graduate Program in Rehabilitation and Functional Performance, Ribeirão Preto Medical School, University of São Paulo, Ribeirão Preto, Brazil; 11 Department of Sport and Sport Science, Exercise and Human Movement Science, University of Freiburg, Freiburg, Germany; 12 Faculty of Health Sciences, Doctoral School of Health Sciences, University of Pécs, 7622 Pécs, Hungary; 13 Digital Development Center, Széchenyi István University, 9026 Győr, Hungary; 14 Department of Otorhinolaryngology-Head and Neck Surgery, University of Pécs Medical School, 7622 Pécs, Hungary

**Keywords:** Aging, Gait, Balance, Dual tasks, Falls

## Abstract

**Background:**

Beam walking is a new test to estimate dynamic balance. We characterized dynamic balance measured by the distance walked on beams of different widths in five age groups of healthy adults (20, 30, 40, 50, 60 years) and individuals with neurological conditions (i.e., Parkinson, multiple sclerosis, stroke, age: 66.9 years) and determined if beam walking distance predicted prospective falls over 12 months.

**Methods:**

Individuals with (n = 97) and without neurological conditions (n = 99, healthy adults, age 20–60) participated in this prospective longitudinal study. Falls analyses over 12 months were conducted. The summed distance walked under single (walking only) and dual-task conditions (walking and serial subtraction by 7 between 300 to 900) on three beams (4, 8, and 12-cm wide) was used in the analyses. Additional functional tests comprised grip strength and the Short Physical Performance Battery.

**Results:**

Beam walking distance was unaffected on the 12-cm-wide beam in the healthy adult groups. The distance walked on the 8-cm-wide beam decreased by 0.34 m in the 20-year-old group. This reduction was ~ 3 × greater, 1.1 m, in the 60-year-old group. In patients, beam walking distances decreased sharply by 0.8 m on the 8 versus 12 cm beam and by additional 1.6 m on the 4 versus 8 cm beam. Beam walking distance under single and dual-task conditions was linearly but weakly associated with age (R^2^ = 0.21 for single task, R^2^ = 0.27 for dual-task). Age, disease, and beam width affected distance walked on the beam. Beam walking distance predicted future falls in the combined population of healthy adults and patients with neurological conditions. Based on receiver operating characteristic curve analyses using data from the entire study population, walking ~ 8.0 of the 12 m maximum on low-lying beams predicted future fallers with reasonable accuracy.

**Conclusion:**

Balance beam walking is a new but worthwhile measure of dynamic balance to predict falls in the combined population of healthy adults and patients with neurological conditions. Future studies are needed to evaluate the predictive capability of beam walking separately in more homogenous populations.

*Clinical Trial Registration Number* NCT03532984.

## Key Points

- Walking balance is normally inferred from walking speed to predict future fall in older adults
- Beam walking distance sharply decreased with age in older versus younger adults
- Beam walking distance decreased further strongly in individuals with neurological conditions
- Beam walking distance (i.e., distance until first balance loss) predicted future falls in older adults and patients with neurological conditions
- Walking ~ 8.0 of the 12 m maximum on low-lying beams predicted future fallers with reasonable accuracy

## Introduction

Dynamic balance refers to an individual’s ability to maintain the vertical projection of the center of mass within the base of support during walking and standing while resisting self- or external perturbations [1]. Dynamic balance, also confined to walking balance in the current report, has been traditionally assessed by “functional tests” with the primary aim of identifying those at risk for mobility disability or falls [2]. However, these tests do not directly measure the dynamic aspect of balance. Already existing functional tests such as the time it takes to cover 4–10 m on a level surface [3], the Timed-Up-and-Go test [4] or the walking component of the Short Physical Performance Battery (SPPB) [5] infer dynamic balance from walking speed without a loss of balance. Even when turns were included in walking tests, instead of balance loss, turning speed was used to predict falls [6]. Other tests, such as the star excursion balance test, the modified Bass test, and the dynamic leap balance test involve unnatural movements or rapid changes of direction—movements seniors never perform [2]. Like performance in static posturography, scores from these functional tests often poorly correlate with dynamic balance (i.e., walking under single and multitask conditions) [7]. In addition, some of these tests are time consuming and need laboratory equipment (e.g., force plates, instrumented walkways) which makes it rather difficult to apply these tests in daily clinical practice. Reactive balance tests (i.e., perturbation-based tests) are often used as a measure of dynamic balance to detect older adult’s risk of falling [8]. However, the relationship between performance in perturbation-based tests and dynamic balance is unclear [9].

Most ‘functional tests’ of dynamic balance thus rely on walking speed to predict fall risk and falls without an actual loss of balance [2, 10, 11]. While walking slower than 1 m/s is associated with fall risk factors in mobility-limited older individuals, walking speed is weakly associated with fall risk factors and inaccurately predicts sub-clinical mobility impairments in healthier individuals [12]. Because ~ 40–50% of falls occur while walking [13] and up to 33% of falls occur to the side probably due to age-impaired mediolateral balance [14], measuring dynamic balance while walking on a narrow, low-lying beam appears a reasonable alternative to ‘functional tests’ relying on walking speed [2, 10, 11, 15–21]. Beam walking could complement linear and circular tandem gait tests that still often end without an actual loss of balance due to censored distance or duration of those tests [22–24]. Beam walking could measure dynamic balance because the reduction in the base of support can strongly but transiently augment instability as the center of mass pivots over the stance leg. Beam walking thus increases the difficulty of postural control and its sensitivity to sub-clinical motor impairments [10]. It ensures a fall-specific, sharp end-point in the form of an actual loss of balance even in older individuals who self-report to be ‘healthy’ and it is an easy-to-administer test [10].

Dysfunctional dynamic balance is a precursor to falls in neurological patients (e.g., multiple sclerosis, stroke) and identifying fall-related risk factors is a priority in health care. Nearly 50% of patients with Parkinson’s disease (PD), multiple sclerosis (MS), and stroke fell once and 32% fell multiple times over 6 months [25]. While the etiology of falls differs between these patient categories, dysfunctional dynamic balance is a shared mechanism underlying the falls these patients experience. Remarkably, disease type and balance confidence but not ‘dynamic balance tests’ or ‘functional tests’ (BBS, Dynamic Gait Index, Timed-Up-and-Go test, 10-m walk test) predicted single and recurrent falls [25], justifying the development of a new walking balance test used in neurological patients as well [2].

The purpose of this preliminary study was to characterize dynamic balance measured by the distance walked on beams of different widths in five age groups of healthy adults (20, 30, 40, 50, 60 years) and individuals with neurological conditions (i.e., PD, MS, stroke). The second aim was to determine if dynamic balance, as measured by beam walking distance, predicts prospective falls over 12 months. Our hypothesis was that age, disease, and beam width affect dynamic balance as measured by distance walked on a narrow, low-lying beam and that beam walking distance predicts future falls, particularly in the older age groups and in individuals with neurological conditions [10, 11].

## Methods

The design of this study has previously been published in the form of a study protocol [2]. Due to the COVID pandemic, instead of a multi-center trial, we deviated from the protocol and were able to conduct only a single-center trial.

### Participants, Design

This is a prospective longitudinal study over 12 months in 97 individuals with neurological conditions (MS, PD, stroke) and 99 healthy adults without neurological conditions. Individuals were recruited in the vicinity of Kaposvár, the seat of Somogy County in western Hungary. There were five age groups of healthy adults (20, 30, 40, 50, 60 years) and one group of patients with various neurological conditions (PD, stroke, MS). Healthy males and females were recruited from public offices and health screening locations according to age categories. A two-step excluding-paradigm was used for participant recruitment. First, a positive answer to any of the following questions in a (phone) interview excluded a healthy participant candidate: inability to walk 10 m independently; knee or hip joint replacements ≤ 6 months before enrollment; uncontrolled cardiovascular disease or angina pectoris; neuromuscular disease; diagnosed Parkinson’s disease, multiple sclerosis, or stroke; cancer therapy ≤ 3 months before enrollment; severe asthma or chronic bronchitis; diagnosed diabetes with neuropathy; poor and uncorrected vision, and a score ≤ 27 on the Mini-Mental State Examination (MMSE) test. All participants had corrected vision. Second, at the start of the laboratory visit, all participants performed the SPPB and those healthy adults with a score ≤ 10 were excluded [5].

Male and female neurological patients were recruited from the hospital’s outpatient day clinic and patient database. Patients who reported with balance and mobility difficulties were eligible. All patients had a medical diagnosis by a neurologist. Patients with PD (Hoehn-Yahr stage 2–3) met the UK Brain Bank criteria. Patients with MS met the McDonald’s criteria of the International Panel on Diagnosis of MS. Patients with stroke met the World Health Organization diagnostic criteria for stroke. Patients with balance disorders visiting the outpatient clinic due to a fall and dizziness were eligible. Exclusion criteria for all participants were: MMSE < 21; major depression (Clinically Useful Depression Outcome score ≥ 46); severe joint and/or bone disorders interfering with balance and gait (clinical judgment); aphasia interfering with comprehension of the aims of the study; MS relapse within 3 months; stroke < 1 month before the start of this study, and phobic postural vertigo. The local ethics committee approved the study protocol and written informed consent was obtained from each participant. The study was conducted according to the Declaration of Helsinki.

### Measurements

The current study focused on the prediction of future falls from dynamic balance performance quantified by beam walking distance and included only selected variables from a large database to characterize the sample [2]. Demographic data included age, sex, height, foot length, foot width, body mass, education, marital and retirement status, and medical diagnosis for patients. Physical activity was estimated by the international physical activity questionnaire that has good reliability (Spearman's rho ~ 0.8). Individuals’ upper extremity function was characterized by the sum of left and right hands’ grip strength measured with a hand-held digital dynamometer (Camry Scale, South El Monte, CA, USA), a measurement with an intraclass correlation coefficient (ICC) of 0.99 in our studies [9]. Lower-extremity function was characterized by SPPB, a composite mobility test. The battery measures balance, 4-m habitual gait speed, and leg strength with good day-to-day reliability (ICC > 0.80) [5]. Dynamic balance was assessed with beam walking under single and dual task conditions [11]. Low-lying aluminum beams were 4 m long, 2 cm high, and 4, 8, and 12 cm wide, covered with slip resistance material, and placed on a thin, black rubber mat. After a practice trial, participants performed 3 trials barefoot on each width with and without a calculation task (subtraction by 7 between 300 to 900) [7]. The order of conditions was fixed: the12cm width was done first followed by the 8 and 4 cm widths with single task first (no calculation). Instructions were: “Traverse the entire length of the beam safely at your preferred speed without stepping off, facing forward, and with your arms folded in front of your chest. Trials end when you step off, walk sideways, or unfold the arms.” Participants were not allowed to grap the beam with the toes. Other than that, foot placement style and speed were not controlled. To reduce the risk for a fall, two technicians walked behind the participants on the floor. Two observers visually observed each trial from each side and measured the length. They immediately marked the spot on the beam where the heel of the foot that remained on the beam after balance was lost. For each beam width, the longest distance walked was entered in the database. As a control condition, we also measured the distance walked in a 4-m-long, 4-cm-wide tape on the floor, i.e., tandem gait, using the same test termination criteria as for beam walking. Global cognition was measured in all participants by the MMSE.

### Recording of Falls

A fall was defined as an event reported either by the faller or by a witness, resulting in a person inadvertently coming to rest on the ground or another lower level, with or without loss of consciousness or injury [26]. Healthy adults over age 20 and all patients (stroke, MS, PD) were asked to fill in a questionnaire concerning falls for the period of 6 months (data not reported here) and 12 months after the start of the study: the number of falls; the day of time of the fall(s); the activity during which the fall(s) had occurred; footwear worn at the time of the fall(s); the location of the fall(s); the mechanism of the fall(s), and the consequences of the falls. Such a fall recall has reasonable validity [26]. We were able to access medical charts in case of a serious outcome. Even if there were no falls, we instructed participants to fill in the questionnaire. Each participant was also contacted by telephone at least once a month to ensure that they kept the fall diaries up to date.

### Statistical Analyses

Data are reported as mean ± SD or median and interquartile range. We compared descriptive characteristics between patients and an age-similar healthy group (i.e., 60-year-old healthy group) using an independent t-test. The 6 groups (5 healthy age decades, 1 patient group) by 2 Tasks (single, dual task) by 3 Beam widths (12, 8, 4 cm) data were analyzed with an analysis of variance (ANOVA) with repeated measures on Tasks and Beam widths followed by a Tukey’s post-hoc contrast when needed. Because beam walking distance is censored (ceiling effect) by the maximal length of 4 m for a given beam width (4, 8, 12 cm) and censored data are not appropriate for linear and logistic regression analyses, we summed the distance walked on the 3 beams (4, 8, 12 cm). The summed beam walking distance data had become not censored, as only 2 (single task) and 7 (dual task) of the 196 individuals walked the maximal distance of 12 m. Using linear regression, we examined the association between dynamic balance as measured by summed beam walking distance with and without a cognitive task and age, sex, type of disease, height, foot length, foot width, body mass, years of education, marital status, retirement status, and fall history separately in healthy adults and patients. Using logistic regression, we predicted prospective falls over 12 months from dynamic balance as measured by beam walking distance in all study participants combined. Finally, we analyzed the predictive capabilities of beam walking distance using receiver operating characteristic (ROC) curves and calculated specificities and sensitivities at the optimal threshold determined by the Youden Index [27]. Using logistic regression and the ROC curves, we also determined the accuracy of predicting falls by beam walking distance for recurrent fallers and various circumstances of falls. The level of significance was set at p < 0.05.

## Results

### Characteristics of the Sample

Table 1 shows the descriptive data of individuals with and without neurological conditions. Included patients had a diagnosis of: PD (n = 18), MS (n = 11), stroke (n = 40), benign paroxysmal positional vertigo (n = 15), phobic postural vertigo (n = 7), and polyneuropathy (n = 6). Across the healthy adult age groups, height, SPPB, physical activity, MMSE, and grip strength tended to be lower and body mass and body mass index tended to be higher with older age. Compared to an age-similar healthy group, patients were not significantly different in age, height (~ 5 cm taller), body mass (2.4 kg lighter), body mass index (~ 2 units lower), SPPB (2 points lower), grip strength (14 kg lower), physical activity (~ 900 points lower score), MMSE (1 point lower), and education years (independent t-test: each p > 0.05).

**Table 1:** Descriptive characteristics of individuals with (patients) and without neurological conditions (healthy adults)

Age decades for healthy adults, y								
Variable	20	30	40	50	60	All	Patients	All
All, n	19	20	20	20	20	99	97	196
Males, n	9	6	9	9	8	41	44	85
Females, n	10	14	11	11	12	58	53	111
Age, y	24.7	34.3	44.6	54.7	64.6	44.8	66.9	55.7
	1.85	2.08	2.14	2.36	2.16	14.33	10.17	16.64
Height, m	174.1	177.1	172.6	175.2	171.7	174.1	176.2	175.1
	4.86	5.86	7.84	6.10	8.60	6.95	6.13	6.62
Foot length, cm	24.7	24.5	25.2	24.9	25.1	24.9	24.9	24.9
	2.21	1.83	1.59	1.92	2.02	1.90	1.99	1.94
Foot width, cm	11.4	11.2	11.3	11.4	11.4	11.4	11.6	11.5
	1.43	1.47	1.11	1.51	1.23	1.33	1.37	1.35
Mass, kg	74.5	77.1	71.9	76.7	76.4	75.3	74.0	74.7
	9.79	8.75	10.14	9.42	8.69	9.39	10.13	9.76
BMI, kg·m^−2^	24.6	24.7	24.2	25.1	26.1	25.0	23.9	24.4
	3.31	3.59	3.24	3.67	4.29	3.63	3.08	3.40
SPPB, score	11.5	12.0	11.9	11.5	11.1	11.6	9.1	10.3
	0.90	0.00	0.37	0.61	0.85	0.70	1.44	1.70
Grip strength, kg	82.8	65.5	68.1	74.3	57.0	69.4	42.9	56.3
	20.45	12.43	17.40	17.30	16.93	18.81	12.71	20.84
IPAQ, score^1^	4467	4308	3634	3716	3329	3885	2442	3171
	1041	871	872	886	770	972	744	1127
MMSE, score	29.0	28.7	29.0	28.4	28.0	28.6	26.9	27.8
	0.88	1.09	0.92	1.23	1.17	1.12	1.55	1.59
Education, y	14.0	14.1	14.8	14.9	13.9	14.3	14.3	14.3
	2.70	1.89	1.94	1.81	2.08	2.10	2.15	2.12
Marital status, n								
Single	8	5	2	1	0	16	1	17
Married	9	14	17	14	18	72	83	155
Divorced	2	0	0	4	1	7	7	14
Widowed	0	1	1	1	1	4	6	10
Retired, n	0	0	0	0	12	12	38	50

Values are mean ± SD (2nd row per variable) or frequencies, n

BMI, body mass index

SPPB, short physical battery performance test

Grip strength, sum of left and right grip strength

IPAQ^1^, international physical activity questionnaire, metabolic equivalent[MET]·min^−1^·week^−1^

MMSE, mini-mental state examination

### Dynamic Balance as Measured by Beam Walking

**Fig. 1:**
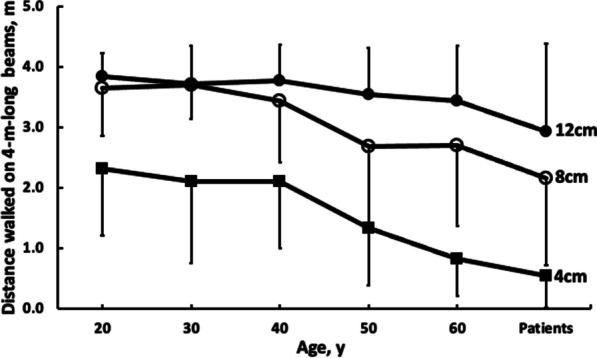
Age and beam width interaction for dynamic balance measured by distance walked on low-lying aluminum beams (length: 4 m, height: 2 cm, widths: 4, 8, 12 cm). With increasing age, beam walking distance decreases. This shortening in distance accelerates after age 40. The distance walked is the shortest in individuals with neurological disease (‘Patients’). These data are pooled across beam walking distances measured with and without a cognitive task. Vertical bars denote ± 1SD

Table 2 shows descriptive values for walking balance as measured by beam walking distance in individuals with and without neurological conditions. Of the relevant and significant interaction effects, the Group by Beam width interaction (F = 2.9, p = 0.002, η^2^ = 0.070) revealed that: a) distance walked became shorter with age (p = 0.011, η^2^ = 0.009); b) with the beam width decreasing, the effect of distance shortening with age became more pronounced (p = 0.041, η^2^ = 0.011), and c) the distance walked was the shortest in patients compared with healthy adults as beam width decreased (p = 0.031, η^2^ = 0.032, Fig. 1). To illustrate, the reduction in distance walked under single-task conditions between 12-cm versus 8-cm wide beam was 0.39 m in healthy adults. However, this reduction was 0.80 m in patients. While significant, the Group by Task interaction revealed only marginal effects across the 6 groups in the distance walked with (dual-task) and without the cognitive task (single-task) (F = 2.8, p = 0.018, η^2^ = 0.069). Cognitive errors during dual-tasking were few and independent of beam width (p > 0.05). Age did not affect distance walked, i.e., tandem gait, on a 4-m-long, 4-cm wide tape (p = 0.823). There was a Group by Time interaction for single and dual task tape-walking (F = 2.5, p = 0.033, η^2^ = 0.061). Due to dual-tasking versus single-tasking, distance walked decreased by up to 1.6 m in the age-groups 50 and 60 compared with the ~ 0.5 m reductions in the other groups (Table 2).

**Table 2:** Walking balance quantified by the distance walked on 4, 8, and 12-cm wide, 4-m long, and 2-cm high low-lying aluminum beams (boards) in individuals with (patients) and without neurological conditions (healthy adults)

Age decades for healthy adults, y								
Variable	20	30	40	50	60	All	Patients	All
12 cm width								
ST distance, 4 m	3.84	3.61	3.81	3.84	3.69	3.76	3.12	3.44
	0.32	0.79	0.56	0.50	0.88	0.64	1.42	1.14
DT distance, 4 m	3.85	3.82	3.74	3.23	3.17	3.56	2.74	3.16
	0.44	0.48	0.63	1.06	0.95	0.80	1.47	1.25
8 cm width								
ST distance, 4 m	3.66	3.95	3.41	2.93	2.90	3.37	2.32	2.85
	0.94	0.13	1.11	1.27	1.31	1.11	1.54	1.44
DT distance, 4 m	3.62	3.45	3.47	2.42	2.49	3.08	1.99	2.55
	0.64	0.99	0.94	1.34	1.36	1.19	1.35	1.38
4 cm width								
ST distance, 4 m	2.34	1.96	1.60	1.24	1.01	1.62	0.56	1.10
	0.86	1.06	1.07	0.66	0.75	1.00	0.47	0.95
DT distance, 4 m	2.30	2.24	2.59	1.40	0.63	1.82	0.50	1.17
	1.36	1.64	1.13	1.23	0.49	1.40	0.55	1.26
Tape, 4 m								
ST	4.00	4.00	3.90	3.89	3.91	3.94	2.88	3.42
	0.00	0.00	0.30	0.32	0.27	0.23	1.48	1.18
DT	3.48	3.51	3.41	2.36	2.93	3.13	2.45	2.80
	0.82	1.02	1.27	1.47	1.37	1.27	1.54	1.45
Total beam distance								
ST distance, 12 m	9.85	9.52	8.83	8.00	7.61	8.75	5.99	7.39
	1.17	1.23	1.47	1.90	2.14	1.81	2.80	2.72
DT distance, 12 m	9.77	9.51	9.80	7.05	6.29	8.47	5.24	6.87
	1.79	2.46	1.82	2.17	2.10	2.54	2.64	3.05

Values are mean ± SD (second row per variable)

12, 8, and 4 cm, denote the width of the 4-m long and 2-cm high aluminum beam

ST, distance walked on the beam without a cognitive dual-task (single task)

DT, distance walked on the beam with a cognitive dual-task (dual task)

Tape, walking on a 4-m long and 4-cm wide tape on the floor

Total distance, sum of distance walked in meters, on the 4, 8, and 12-cm wide beams

Light grayed portion, data included in the regression analyses (healthy adults) and the data denoted by light and dark grayed portions combined, were included in the logistic regression (individuals with and without neurological conditions)

### Association Between Dynamic Balance as Measured by Beam Walking and Anthropometric and Demographic Data

Based on data in healthy adults (n = 99), beam walking performance without a cognitive task was linearly but weakly associated with age (F = 25.9, p = 0.001, R^2^ = 0.21, y = − 0.06x + 11.4) and this association was similar under dual-tasking (F = 35.6, p = 0.001, R^2^ = 0.27, y = − 0.09 + 12.6). These continuous data analyses confirm the analyses of the categorical data using analysis of variance presented above. Beam walking distance was not associated with sex, height, foot length, foot width, body mass, educational status, marital status, or retirement status.

Based on data in patients (n = 97), beam walking performance without a cognitive task was linearly but weakly associated with sex and years of education (F = 6.1, p = 0.003, R^2^ = 0.12.

y = − 1.3(sex) − 0.30(years of education) + 10.9). While dual-tasking, beam walking performance was linearly but weakly associated with sex only (F = 5.8, p = 0.018, R^2^ = 0.05, y = − 1.3x + 5.8). Beam walking distance was not associated with age, height, foot length, foot width, body mass, marital status, or retirement status. Therefore, beam walking distance was not normalized for foot length and foot width in either group.

### Number and Circumstances of 12-Months Prospective Falls

Table 3 summarizes the number and circumstances of prospective falls in individuals with and without neurological conditions for the 12 months after the start of the study. In total, 122 out of 196 individuals (62%) experienced 423 falls over 12 months. Over 12 months, 27% of the 99 healthy adults experienced a fall, resulting in a total of 69 falls. No falls occurred in the 20-year-old participants, and there were 3, 7, 7, and 10 individuals who reported falling in the 30, 40, 50, and 60 age-decade, respectively. Over 12 months, 98% of the 95 patients reported falling, resulting in a total of 354 falls.

**Table 3:** Summary of prospective falls data and circumstances of falls in individuals with (patients) and without neurological (healthy adults) conditions for the 12-months follow-up period

Variable	Healthy adults	Patients	All
N	99	97	196
Persons fell, n	27	95	122
Number of falls: 1–2x	54	143	197
Number of falls: 3–6x	15	211	226
All falls, n	69	354	423
Day of time			
Morning	30	128	158
Afternoon	18	108	126
Evening	21	117	138
Activity when falling			
Walk	12	58	70
Rise, step, turn	34	173	208
Sit, lean, other	23	122	145
Footwear			
Firm	42	191	233
Loose	27	163	190
Location			
Outdoors	43	185	228
Indoors	26	169	195
Mechanism			
Balance loss, dizziness	25	97	122
Knees buckling, weakness	28	153	181
Turn, step, bump	16	105	120
Consequence of falls			
Fractures, joint injury	18	51	69
Skin damage	95	259	354

The grayed data were used in the logistic regression analyses

Skin damage denotes excoriation, contusion, abrasion, cuts, other

In both healthy adults and the patient groups, ~ 40% or 128 of the falls occurred in the morning (Table 3). In both groups, ~ 50% of falls occurred while rising to a higher position from a lower position, stepping up or down, or while turning. Only ~ 16% of falls occurred while walking. Nearly 60% of falls occurred while wearing firm versus loose footwear. In the two groups, most falls occurred outdoors (healthy adults 62%; patients: 53%). In each group, ~ 40% of falls was due to knees buckling or weakness. Falls caused fractures or joint dislocations (healthy adults: 49%, patients: 33%) or excoriation, contusion, abrasion, or cuts (healthy adults: 51%, patients: 67%). In healthy adults, 26% of falls caused fractures or joint dislocations compared with 14% of falls in patients.

### Prediction of Falls by Dynamic Balance as Measured by Beam Walking Distance

The logistic regression demonstrated that the total distance (i.e., all three beams combined) and distances walked on a single beam (4-, 8-, or 12-cm wide) significantly predicted the risk of 12-month prospective falls (Table 4). In the ROC analysis, total distance (both single- and dual-task) and single-task distance on the 4-cm wide beam demonstrated the best predictive capabilities with the area under the curve (AUC) between 0,74 and 0,76 (Table 4).

**Table 4:** Predictive capabilities of beam walking for prospective falls at 12 months

Test variant	Coef	SD	p-value	AUC	Threshold	Specificity	Sensitivity
Total, single	− 0.38	0.08	< 0.001	0.74	9.0	0.54	0.84
Total, dual	− 0.36	0.06	< 0.001	0.76	8.2	0.66	0.75
4-cm, single	− 0.97	0.19	< 0.001	0.75	1.0	0.68	0.72
4-cm, dual	− 0.66	0.13	< 0.001	0.72	0.7	0.66	0.75
8-cm, single	− 0.42	0.12	< 0.001	0.64	2.9	0.78	0.50
8-cm, dual	− 0.61	0.13	< 0.001	0.71	2.9	0.74	0.66
12-cm, single	− 0.50	0.17	0.005	0.57	2.0	0.97	0.22
12-cm, dual_	− 0.54	0.15	< 0.001	0.62	2.0	0.95	0.30

The ‘Test variant’ includes the combined (total) distance walked on all three beams and the distances walked on individual beams (4-, 8-, or 12-cm wide), either as a single task or a dual task with an added cognitive task. The table combines results from logistic regression (columns Coef, SD, p-value) and Receiver Operating Characteristic (ROC) analysis (columns AUC, Threshold, Specificity, Sensitivity). Coef: beta-coefficient from logistic regression. AUC: Area Under the Curve

**Fig. 2:**
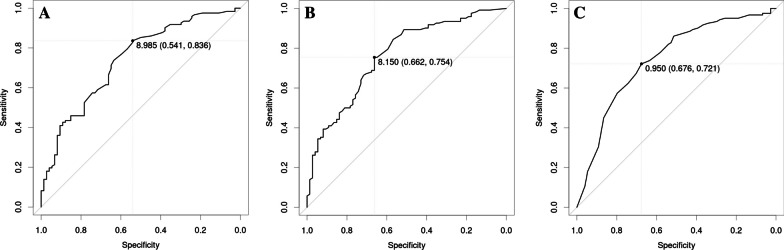
Receiver operating characteristic (ROC) curves for predicting prospective falls over 12 months in individuals with and without neurological conditions (n = 196), from dynamic balance measured by total beam walking distance without (**A**) and with (**B**) a concurrent calculation dual task, and distance walked only on the 4-cm wide beam without the dual task (**C**). There was no difference between the ROCs. The single point marked on the ROC curve indicates the threshold distance (in meters) for optimal specificity and sensitivity determined by the Youden index. The numbers in brackets indicate specificity and sensitivity coupled with this threshold. The manuscript text accompanying the ROCs gives further details

The ROC analyses further revealed that the optimal threshold of 9.0 m (area under the curve, AUC 0.74) distance walked on the beam without a cognitive task was coupled with high sensitivity (0.84) and poor specificity (0.54). For the dual-task condition, at a threshold of 8.2 m walked (AUC 0.76) was coupled with reasonable specificity (0.66) and sensitivity (0.75). Similar specificity (0,68) and sensitivity (0.72) was coupled with a threshold of 1.0 m walked on the 4-cm wide beam under the single-task condition (Table 4, Fig. 2). Compared with these data, predictive capabilities of beam walking distance did not improve for single versus recurrent falls or for various circumstances of falls (data not shown).

## Discussion

We characterized dynamic balance measured by the distance walked on 4-, 8-, and 12-cm-wide beams under single- and dual-task conditions in individuals with and without a neurological condition. We also determined if dynamic balance would predict prospective falls over 12 months. We found that age, disease, and beam width affect dynamic balance as measured by distance walked on narrow, low-lying beams and that beam walking distance predicts future falls.

### Characterization of Dynamic Balance as Measured by Beam Walking Distance

In agreement with our study hypothesis, age, disease, and beam width all affected dynamic balance as measured by the distance walked on low-lying beams (Table 2, Fig. 1). Beam walking distance was unaffected on the 12-cm-wide beam in the five age groups of healthy adults (3.76/4.00 m). However, the distance walked on the 8-cm-wide beam decreased by 0.34 m already in the 20-year-old group. This reduction was ~ 3 × greater, 1.1 m, in the 60-year-old group. In contrast to these large reductions, the distance walked during tandem gait over a 4-cm-wide tape on the floor was unaffected. Thus, beam walking versus tandem-walking on the floor represents a different and challenging balance task most likely due to the widths of the beams. Reductions in distance walked on the beams suggest the presence of sub-clinical impairments in the abilities that control walking balance. These impairments seem to remain undetected by standard balance tests such as tandem-walking on a tape glued to the floor (4.0/4.0 m) or by the frequently used SPPB (11.6/12.0, Tables 1, 2). The additional and large reductions in beam walking distance on the narrowest, 4-cm-wide beam point to a floor effect: this condition is too difficult for even healthy adults age 20–60. The potentially greater sensitivity of beam walking versus tandem-walking to detect subtle impairments in walking balance could be related to a reduction not only in the base of support (i.e., distance between the two feet) but to the reduction also in the contact area at the interface between the feet and the board. Such a mechanical constraint can strongly but transiently augment instability as the center of mass pivots over the stance leg. Instability increases during beam-walking but less so during tape-walking because even during normal gait, the path of the center of mass travels outside the medial border of the supporting foot [28] especially in old adults [29]. Thus, the path of the center of mass passes close to the beam edge, making older individuals ‘feel’ that they could lose their balance and they step off the beam (‘fall’). This sense of imminent balance loss reduces beam-walking distance. Beam walking thus increases the difficulty of postural control and its sensitivity to sub-clinical motor impairments. It ensures a fall-specific, sharp end-point in the form of an actual loss of balance even in older individuals who self-report to be ‘healthy’ [2, 10]. In total, these data seem to suggest that an optimal beam width probably lies around 6-8 cm that could avoid floor and ceiling effects for a new balance test to be established. Our data imply that beam walking could complement or even replace certain ‘functional tests’ currently in use to measure walking balance based on walking speed without an actual loss of balance [23, 24].

Neurological diseases strongly affected beam walking distance and much more so than it affected SPPB (9.1/12 points) (Table 2, Fig. 1). Some studies actually suggest that a score of 9 on the SPPB is a ‘high performance’. Contrasting with these SPPB scores, beam walking distances decreased sharply by 0.8 m on the 8 versus 12 cm beam and by additional 1.6 m on the 4 versus 8 cm beam. The 0.53 m distance walked by patients on the 4-cm-wide beam suggests a floor effect: the task was extremely difficult. An interesting observation was that patients walked numerically identical distances, i.e., 2.9 m, on the 12-cm-wide beam and the 4-cm-wide tape glued to the floor. These data suggest that walking on a wide beam may not provide additional benefits over tandem walking but could provide additional insights into walking balance over ‘functional tests’ (SPPB, walking speed) in patients we examined in the present study. Because dysfunctional walking balance is a precursor to falls in neurological patients, an accurate identification of fall-risk factors remains a priority in this population and beam walking might be an effective adjuvant to ‘functional tests’ currently in use in such patients [25].

Beam walking with a cognitive dual task did not significantly reduce the distance walked on the three beams (Table 2). These data are unexpected and in contrast with a previous study that reported strong effects of cognitive dual-tasking on beam-walking distance [11]. One would expect that when the motor task is difficult and demands attention, adding a secondary cognitive task would strongly reduce motor performance. In that study older individuals were ~ 6 years older than our participants in the 6th decade and they walked significantly slower when dual-tasking on the beams. Because in the present study the number of errors (1.0–1.5) while dual-tasking did not differ between age groups and beam widths, individuals perhaps prioritized the motor element of motor-cognitive dual-tasking. Our patients walked on the tape ~ 1 m shorter distance (2.9 m) than age-similar healthy adults (3.9 m). The tape-walking performance was already so low that dual-tasking had little potential to reduce it further (p > 0.05; reduction of 0.4 m, Table 2). Additional data are needed to confirm the effects of cognitive dual-tasking on walking balance. This is because adding a secondary cognitive task to the Timed-Up-and-Go test did not increase the accuracy of fall prediction [30]. Therefore, the role of cognitive dual-tasking in walking balance remains unclear.

### Incidence and Circumstances of Falls

Table 3 shows that there were 122 individuals with 423 falls. Over 80% of these falls occurred in patients during the 12-month-long follow-up period. Some previous studies reported falls in healthy young individuals age 20 even after excluding sports-related falls [31]. Admittedly, our study has low sample sizes in the age-decades (Table 1), but we did not observe a single fall in the 20-yearl-old participants (n = 19). However, 15%, 35%, 35%, and 50% of individuals reported falling in the 30-, 40-, 50-, and 60-year age-decade, respectively. These data agree with the 30–40% rates reported previously for the corresponding age-brackets in ~ 25,000 community dwelling US adults [32].

There were only 2 individuals with a neurological condition who did not report falling. Our 98% fall rate is twice as high as the 47% rate for those who reported falling 1–2 × and the 60% proportion of patients reporting 3–6 × recurring falls is also ~ twofold greater than the 32% rate reported previously also in individuals with PD, MS, and stroke diagnoses [25]. We had one patient who reported falling six times over 12 months. The age and sex distribution of patients were similar to those in Italy [25]. The smaller sample size, perhaps the higher level of impairment and the lower quality of outpatient care contributed to the high rate of (recurrent) falls in our study.

In age-decades 40–60, most falls occurred outdoors in the morning, which is probably related to why most falls occurred while wearing firm shoes. Most frequently falls occurred due to the knees buckling or weakness. Our older adults and patients had very low grip strength and low strength and muscle mass are related to falls [33] but this association is not always present and requires further confirmation [34, 35]. Our data contrast with reports suggesting that ~ 40–50% of falls occur while walking [13], as we observed that ~ 50% of falls occurred while rising to a higher position from a lower position, stepping up or down, or while turning in standing in individuals with and without a neurological condition (Table 3). Because the amount of physical activity based on the International Physical Activity Questionnaire or IPAQ scores was ~ 1,000 units higher in our older individuals than in some other studies [36], the reason for the low fall incidence during walking, even in our patient group, remains unclear. Indeed, the association between physical activity, sedentary behavior, and falls is complex. Being up on one’s feet and being physically active naturally increases the potential for a fall to occur. However, high levels of chronic physical activity can at the same time improve fitness, which is known to reduce falls risks [37, 38]. Improving some of these environmental risk factors for falls can also reduce risks for and incidence of falls [39].

### Correlates of Beam Walking Performance and Prediction of Future Falls

There is a strong effort underway to identify tests that are associated with falls risks and incidence of falls through the age and disease spectrum [39–46]. Of the commonly examined variables such as age, sex, fall history, body fat, education, marital status, or retirement status, beam walking distance was only associated with age which in turn predicted falls over 12 months in healthy adults aged 20–60 years. These associations and predictions were independent of performing beam walking under single or dual task condition. Our findings complement prior data suggesting inconsistent, weak, or even no associations between risk factors for falls and incidence of falls. While many studies suggest that lower extremity muscle strength and power are associated with balance, fall risks, and future falls, there is also evidence suggesting to the contrary with no such associations [42]. A systematic review found that none of the biomechanical markers of challenging walking tasks correlated with fall risk variables and fall prediction was inaccurate without including fall history [40]. Indeed, our data suggest that performance in a difficult walking balance task is associated with age and predicts future falls without fall history. The logistic regression coefficient is reasonably large (coefficient: = − 0.38 ± 0.08, p = 0.001) but dual-task condition did not improve prediction accuracy. The coefficient of − 0.38 means that odds of fall (i.e., ratio fall/no fall) change 0.68 time (e^− 0.38 = 0.68): with each increase in distance walked by 1 m, the odds decrease by 0.32 (1–0.68). With additional meters walked, the effect is multiplicated: for a difference of, e.g., 4 out of 12 m, the odds change 0.68^4 = 0.21 time. That is, the odds decrease by nearly 80%: the odds of fall/no fall would move from 6/6 to 2/10. We interpret these data as clinically meaningful.

Figure 2 shows that dual-task beam walking distance of ~ 8 of 12 m maximum (AUC 0.76) was coupled with specificity (0.66) and sensitivity (0.75). Similar specificity and sensitivity was achieved using the threshold of 1.0 m walked on the 4-cm wide beam under the single-task condition. Using just one beam instead of three, without the need for the added cognitive task, seems like a plausible alternative when using the test in clinical settings. These data imply that based on beam walking distance we would miss to identify many of those who would eventually fall and would erroneously identify many individuals as fallers even though they would actually not experience a fall. In patients, sex and education emerged as correlates of beam walking distance, agreeing with a previous report [43]. In contrast to this report’s finding, we found no evidence that the fall risk varies among different disease types. We did not examine or find no associative or predictive role in falls several health conditions (vision, depression, arthritis, alcohol) or functional limitations (ability to climb stairs or perform daily functions) [41, 46].

### Limitations

The current study did not compare how accurately conventional ‘functional tests’ versus dynamic balance measured by beam walking distance predicted future falls. This will be reported in a future study. Our data are limited by the homogeneity of fall incidence in patients, i.e., virtually all patients reported falling. Consequently, the combined analysis of individuals with and without neurological conditions, while necessary to ensure a balanced representation of fallers and non-fallers, presents a limitation as it may not distinctly separate the impact of dynamic balance impairments from other disease-related mechanisms in fall risk, particularly given the high incidence of falls among participants with neurological conditions. This preliminary and exploratory study cannot provide definitive ‘normative data’ for beam walking distances and clear cutoffs of ‘low’, ‘medium’, and ‘high’ levels of dynamic balance by age and sex due to low samples sizes. While gait analysis with wearables can identify age groups and retrospective falls highly accurately by analyzing dynamical systems outcomes with machine learning, such approaches require large sample sizes and sophisticated algorithms and still miss individual cases, leaving room for ‘analog’ solutions such as beam walking [47–49].

## Conclusion

Dynamic balance assessed by the distance walked on low-lying beams is associated with age in healthy adults and predicts future falls over 12 months in the combined population of healthy individuals and patients with neurological conditions. Additional studies are needed to determine the viability of walking on a balance beam to become a new measure of dynamic balance to predict falls across the spectra of age and disease.

## Data Availability

The datasets generated during and/or analyzed during the current study are available from the corresponding author on reasonable request.

## Abbreviations

ANOVA: Analysis of variance
AUC: Area under the curve
BBS: Berg balance scale
BESTest: Balance evaluation systems test
CA: California
COVID: Corona virus disease
ICC: Intraclass correlation coefficient
IPAQ: International physical activity questionnaire
ROC: Receiver operating characteristic
PD: Parkinson’s disease
(mini)BESTest: Mini balance evaluation systems test
MMSE: Mini-mental state examination
MS: Multiple sclerosis
SD: Standard deviation
SPPB: Short physical performance battery
UK: United Kingdom
USA: United Stated of America
